# Rd9 Is a Naturally Occurring Mouse Model of a Common Form of Retinitis Pigmentosa Caused by Mutations in RPGR-ORF15

**DOI:** 10.1371/journal.pone.0035865

**Published:** 2012-05-01

**Authors:** Debra A. Thompson, Naheed W. Khan, Mohammad I. Othman, Bo Chang, Lin Jia, Garrett Grahek, Zhijian Wu, Suja Hiriyanna, Jacob Nellissery, Tiansen Li, Hemant Khanna, Peter Colosi, Anand Swaroop, John R. Heckenlively

**Affiliations:** 1 Department of Ophthalmology and Visual Sciences, University of Michigan Medical School, Ann Arbor, Michigan, United States of America; 2 Department of Biological Chemistry, University of Michigan Medical School, Ann Arbor, Michigan, United States of America; 3 Jackson Laboratory, Bar Harbor, Maine, United States of America; 4 Neurobiology-Neurodegeneration & Repair laboratory, National Eye Institute, National Institutes of Health, Bethesda, Maryland, United States of America; 5 Department of Ophthalmology, University of Massachusetts Medical School, Worcester, Massachusetts, United States of America; Massachusetts Eye & Ear Infirmary, Harvard Medical School, United States of America

## Abstract

Animal models of human disease are an invaluable component of studies aimed at understanding disease pathogenesis and therapeutic possibilities. Mutations in the gene encoding retinitis pigmentosa GTPase regulator (RPGR) are the most common cause of X-linked retinitis pigmentosa (XLRP) and are estimated to cause 20% of all retinal dystrophy cases. A majority of *RPGR* mutations are present in ORF15, the purine-rich terminal exon of the predominant splice-variant expressed in retina. Here we describe the genetic and phenotypic characterization of the retinal degeneration 9 (Rd9) strain of mice, a naturally occurring animal model of XLRP. Rd9 mice were found to carry a 32-base-pair duplication within ORF15 that causes a shift in the reading frame that introduces a premature-stop codon. *Rpgr* ORF15 transcripts, but not protein, were detected in retinas from Rd9/Y male mice that exhibited retinal pathology, including pigment loss and slowly progressing decrease in outer nuclear layer thickness. The levels of rhodopsin and transducin in rod outer segments were also decreased, and M-cone opsin appeared mislocalized within cone photoreceptors. In addition, electroretinogram (ERG) a- and b-wave amplitudes of both Rd9/Y male and Rd9/Rd9 female mice showed moderate gradual reduction that continued to 24 months of age. The presence of multiple retinal features that correlate with findings in individuals with XLRP identifies Rd9 as a valuable model for use in gaining insight into ORF15-associated disease progression and pathogenesis, as well as accelerating the development and testing of therapeutic strategies for this common form of retinal dystrophy.

## Introduction

Retinitis pigmentosa (RP) is a group of clinically and genetically heterogeneous progressive retinal degenerative disorders that are characterized by rod and cone photoreceptor dysfunction and death, and which often culminate in blindness [1]. X-linked forms of RP (XLRP) are among the most severe [2], comprising an estimated 15% of total non-syndromic, non-systemic cases [3], [4]. Six genetic loci have been mapped for XLRP; of these, *RP3* is the predominant subtype (www.sph.uth.tmc.edu/retnet/). *RP3* is associated with mutations in the gene encoding Retinitis Pigmentosa GTPase Regulator (RPGR), which encodes multiple alternatively-spliced forms that all share a common amino-terminal domain with homology to Regulator of Chromosome Condensation 1 (RCC1) [4], [5]. The major form of RPGR detected in most tissues (the constitutive form) is produced by exons 1 to 19, whereas the predominant form of RPGR in retina results from an alternatively-spliced transcript containing a unique terminal exon, ORF15. Mutations in ORF15 account for the majority of *RP3* cases, and thus, this region is considered to be a mutation ‘hotspot’ [3]–[5]. To date, all of these mutations are deletions or duplications of purine-rich repeats in ORF15 that produce shifts in the reading frame which predict premature termination of translation.

*RPGR* mutations are often associated with rod-cone degeneration that is highly variable in degree of severity. Affected males generally show signs of night blindness by the first decade of life, progressing to vision loss by the fourth decade [6]–[9]. Female carriers can at times exhibit clinical findings, including electroretinogram (ERG) defects [6], [7], [10]. In some instances, *RPGR* mutations are associated with cone-rod degeneration, atrophic macular degeneration, and syndromic phenotypes [4], [11]–[13].

The constitutive form of RPGR includes a C-terminal isoprenylation sequence and is detected in Golgi complex [14], whereas RPGR-ORF15 localizes to centrosome in cultured cells [15] and predominantly to the transition zone of the photoreceptor cilium [16], [17]. RPGR can interact or exist in complexes with multiple transport and/or ciliopathy-associated proteins, including RPGRIP1, RPGRIP1L, NPHP5 and CEP290 [17]–[23].

To understand the pathogenesis of retinal degeneration, animal models have been used to study RPGR function. *Rpgr*-knockdown in zebrafish resulted in severe developmental abnormalities, including those associated with ciliary function [24], [25]. However, complete loss of Rpgr protein in knockout mice (*Rpgr*-KO), generated by deletion of exons 4 to 6, led to a relatively slow photoreceptor degeneration phenotype [26]. A second strain of mutant mice, generated by deletion of *Rpgr* exon 4, exhibited a variable phenotype depending on the genetic background, resulting in either rod- or cone-dominated disease [27]. Two canine mutants (XLPRA1 and XLPRA2) resulting from nonsense and frame-shift mutations in *RPGR* ORF15, respectively, demonstrated distinct patterns of progression of X-linked retinal atrophy [28]. These canine models have provided an excellent platform to study disease progression and evaluate the efficacy of gene-replacement therapy [29]; however, corresponding mouse models with a relatively shorter generation time have the potential to significantly accelerate progress in defining RPGR-ORF15 function, as well as the cell biological and biochemical bases of the disease associated with ORF15 mutations. Combining studies in mice with those in other animal models is thus predicted to rapidly advance understanding of the phenotypic variations and pathogenesis associated with *RPGR* mutations, as well as the effectiveness of novel therapeutic strategies.

We now describe the genetic, phenotypic and biochemical characterization of Rd9 mice that were previously identified among the strains curated by The Jackson Laboratory as a naturally occurring model of X-linked retinal degeneration present on the C57BL/6J (B6) background [30]. We have identified a disease-associated mutation in mouse *Rpgr* ORF15 that is similar to mutations identified in many XLRP patients. Rd9/Y male mice do not express the *Rpgr*-ORF15 isoform, which in turn affects the expression of other photoreceptor proteins. We also describe in detail the Rd9 retinal phenotype. Our studies suggest that Rd9 is an excellent mouse model for investigating the retinal degeneration caused by ORF15 mutations, as well as for developing gene-based and other therapies.

## Materials and Methods

### Animals

Experimental procedures involving animals were performed in accordance with the guidelines and under the approval of the University Committee on Use and Care of Animals at the University of Michigan, the Institutional Animal Care and Use Committee at The Jackson Laboratory, and the Animal Care and Use Committee at the National Eye Institute; and were in compliance with the statement for ethical care and use of animals of the Association for Research in Vision and Ophthalmology (ARVO). Mice were raised in 12-hour on/12-hour off cyclic lighting. For linkage mapping, extensive backcrosses were carried out between (CAST/Rd9)F1 and C57BL/6J, resulting in the generation of over 360 meioses with different haplotypes. Fundus photography was performed after pupil dilation with 1% atropine, and images were captured using a Genesis small animal fundus camera (Kowa, Torrance, CA) fitted with a 90-diopter condensing lens.

### PCR and Sequence Analysis

Genomic DNA was extracted from tail snips and retinas from Rd9 and wild-type C57BL/6J (B6) mice using Puregene reagents (Gentra, Minneapolis, MN). Total retinal RNA was isolated using RNAqueous (Ambion, Austin, TX) and first strand cDNAs were generated using oligo-dT primers and Superscript II (Invitrogen, Carlsbad, CA). RT-PCR amplifications were performed using the Expand Long Template PCR system (Roche, Indianapolis, IN) and *Rpgr*-specific primers (see Table S1 for all primer sequences). For sequencing *Rpgr* exons 1–19, the cDNAs were PCR amplified and gel-purified products were sequenced using the same primers. For sequencing exon ORF15, genomic DNAs were amplified using AccuPrime high fidelity Taq polymerase (Invitrogen, Carlsbad, CA) and Rap3 and P7 primers, and the 2.2 kb gel-purified products were sequenced using the same primers, as well as the internal primers F208 and R1476.

### Immunoblot Analysis

Rod outer segments (ROS) from male Rd9/Y mice (1 month-of-age) in room light were prepared by gradient centrifugation [31]. Extracts of total retinal proteins were generated by homogenizing dissected retinas in phosphate buffered saline (PBS) plus protease inhibitors, and cellular debris was removed by centrifugation. Protein concentrations were determined using Micro BCA assay kit (Pierce, Rockford, IL). Immunoblot analysis was performed using standard methods with primary antibodies against RPGR [26], [32], rhodopsin [33], transducin-alpha subunit (GeneTex, Irvine, CA), arrestin (ABR AffinityBioreagents, Golden, CO), and recoverin (Chemicon, Billerica, MA), and reactivity was visualized using alkaline phosphatase-conjugated secondary antibodies and reagents (Sigma-Aldrich, St. Louis, MO).

### Immunohistochemistry and Retinal Histology

For immunohistochemical analysis, mice were anesthetized and perfused with 4% paraformaldehyde, the eyes were enucleated and transitioned to sucrose:OCT, and flash frozen. Cryosections (10 µm) were incubated with primary antibodies against rhodopsin [33], S-opsin and M-opsin (Chemicon, 1∶500), and labeling was visualized using goat anti-rabbit AlexaFluor-488 secondary antibody and standard methods [34]. For analysis of retinal histology, anaesthetized mice were perfused with 2% paraformaldehyde plus 2% glutaraldehyde, the eyes were enucleated and post-fixed at least 1 h, and then dehydrated and embedded in JB-4 plastic. Sections (5 µm) were stained with Lee’s stain, and imaged on a fluorescence microscope with digital camera. Measurements of retina layer thickness were made on sections parallel (superior, inferior) and orthogonal (nasal, temporal) to the vertical meridian of the eye, and were plotted vs. distance from the optic nerve head [35]. The statistical significance of differences between the two groups (B6 vs. Rd9) was evaluated by Wilcoxon and t-tests using mixed linear regression.

### Electroretinography

ERGs were performed as described previously [36], [37] using the Espion e^2^ recording system (Diagnosys, Lowell, MA). Briefly, mice were dark-adapted overnight and anesthetized with an intra-peritoneal injection of Ketamine (93 mg/kg) and Xylazine (8 mg/kg). Pupils were dilated with topical atropine (1%) and tropicamide (0.5%). Body temperature was maintained at 37°C with a heating pad. Corneal ERGs were recorded from both eyes using gold wire loops with 0.5% tetracaine topical anesthesia and a drop of 2% methylcellulose for corneal hydration. A gold wire loop placed in the mouth was used as reference, and a ground electrode was on the tail. The ERG protocol consisted of recording dark-adapted ERGs to brief white flashes from −5.8 to +1.09 log cd.s.m^–2^/flash in steps of 0.5 log units. Responses were amplified at 1,000 gain at 1.25 to 1000 Hz, and digitized at a rate of 2000 Hz. A notch filter was used to remove 60 Hz line noise. Responses were computer averaged and recorded at 3 to 60 s intervals depending upon the stimulus intensity. Light-adapted ERGs were recorded after 10 minutes of adaptation to a white 32 cd.m^–2^ rod-suppressing background. ERGs were recorded for stimulus intensities from –0.91 to +1.09 log cd.s.m^–2^ over a 2 log unit range in steps of 0.3 log units. A white 2.0 log cd.s.m^–2^ was used to record the photopic negative response (PhNR), which originates from the ganglion cells [38], [39]. ERG analysis was performed on Rd9/Y male mice (n = 56, ages 1 to 24 months), Rd9/Rd9 female mice (n = 15, ages 1 to 20 months), and wild-type B6 mice (n = 35, ages 1 to 24 months).

## Results

### Rd9 Fundus Features

Rd9 mice were originally identified as a naturally occurring retinal degeneration strain on the basis of pathological findings evident on indirect ophthalmoscopy. Comparison of images from Rd9 and wild-type B6 mice showed unique changes in Rd9/Y male mice at ages as young as 5 months old; these included retinas with “creamy blonde" appearance and pronounced loss of pigment (Figure S1, top). Fluorescein angiography showed telangiectasia and some leakage from vessels (Figure S1, bottom). Fundus photographs of Rd9/Rd9 females appeared similar to those of Rd9/Y males, while photographs of Rd9/+ females showed only diffuse white spots.

### Genetic Analysis and Identification of *Rpgr*-ORF15 Mutation in Rd9

**Figure 1:**
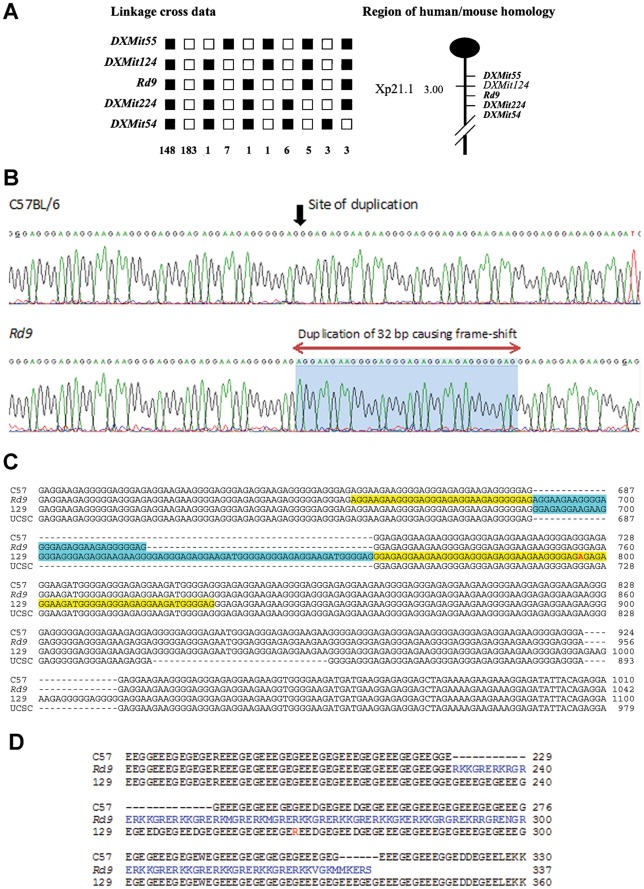
Identification of the genetic defect on the X-chromosome in Rd9 mice. **A**) Haplotype analysis showing the segregation patterns of Rd9 and flanking markers in (CAST/Rd9)F1 x B6 backcrosses generating 364 meioses. Each column represents the chromosome inherited by a group of backcross progeny, with black boxes representing BB homozygotes and white boxes denoting BC heterozygotes for a given locus. The number of offspring that inherited the haplotype of that region of the X Chromosome is listed at the bottom of each column. Also shown is the partial chromosome linkage map with the positions of Rd9 and its flanking markers. **B**) Chromatograms showing partial genomic sequence of *Rpgr*-ORF15 in B6 and Rd9 mice, showing the location of a 32 bp duplication in the Rd9 strain. The precise location is difficult to define because of the highly repetitive nature of the sequence. **C**) Comparison of *Rpgr*-ORF15 genomic sequences of B6, Rd9, and 129/SvJ mice determined by DNA sequence analysis and aligned using ClustalW2. The B6 genomic sequence of *Rpgr*-ORF15 from the UCSC genome browser is also included. Additional tandem duplications found in the Rd9 and 129/SvJ sequences are highlighted in yellow and blue**.** D) Alignment of Rpgr-ORF15 amino acid sequences derived by translation of the B6, Rd9, and 129/SvJ genomic sequences, and the sequence of B6 from the UCSC Genome Browser. The sequences were aligned using ClustalW2.

Extensive backcrosses of Rd9 mice ((CAST/Rd9)F1×B6), combined with genotyping of 364 informative meioses, mapped the Rd9 mutation to the X-chromosome between markers *DXMit55* and *DXMit224* (Figure 1A), a region syntenic with the human Xp21.1 (XLRP-*RP3-RPGR)* locus. Analysis of the constitutively-expressed *Rpgr* isoform (exons 1–19) revealed no sequence change between Rd9 and wild-type B6 mice. However, sequence analysis of the alternatively-spliced *Rpgr* exon ORF15 identified a 32-bp duplication in Rd9 genomic DNA that was not present in B6 DNA (Figure 1B and 1C). Due to the repetitive nature of the sequence, the exact position of the 32-bp duplication is difficult to assign. In the Rd9 ORF15 sequence, six 32-bp repeats are present. For the first five, these repeats are separated by a 4-nucleotide sequence. However, the sixth repeat begins immediately following the end of the fifth repeat, suggesting that the duplication is after position 687 of ORF15, as shown in Figure 1C. The duplication produces a frame shift in the repetitive region of ORF15, predicting a truncated protein in which the C-terminal 108 amino acids are unrelated to the wild-type Rpgr protein and are predominantly basic (61%) (Figure 1D).

The ORF15 sequence from another wild-type strain, 129/SvJ, was also examined for comparison to Rd9. The 129/SvJ sequence has an in-frame 72-bp duplication and an in-frame insertion compared to the B6 sequence; however, again, precise placement of the duplication and insertion is not possible because of the repetitive nature of this sequence (Figure 1C).

The sequence of ORF15 from C57BL/6 present in the UCSC Genome Browser was found to be 31 nucleotides shorter than our ORF15 sequence from B6 (Figure 1C). This 31-bp deletion would cause a frame-shift and result in a severely truncated *Rpgr* protein. The deletion may represent an intra-strain variant, or a sequencing error in the UCSC C57BL/6 sequence, an issue that will require further studies to resolve.

### *Rpgr* mRNA and Protein Expression in Rd9 Retina

**Figure 2:**
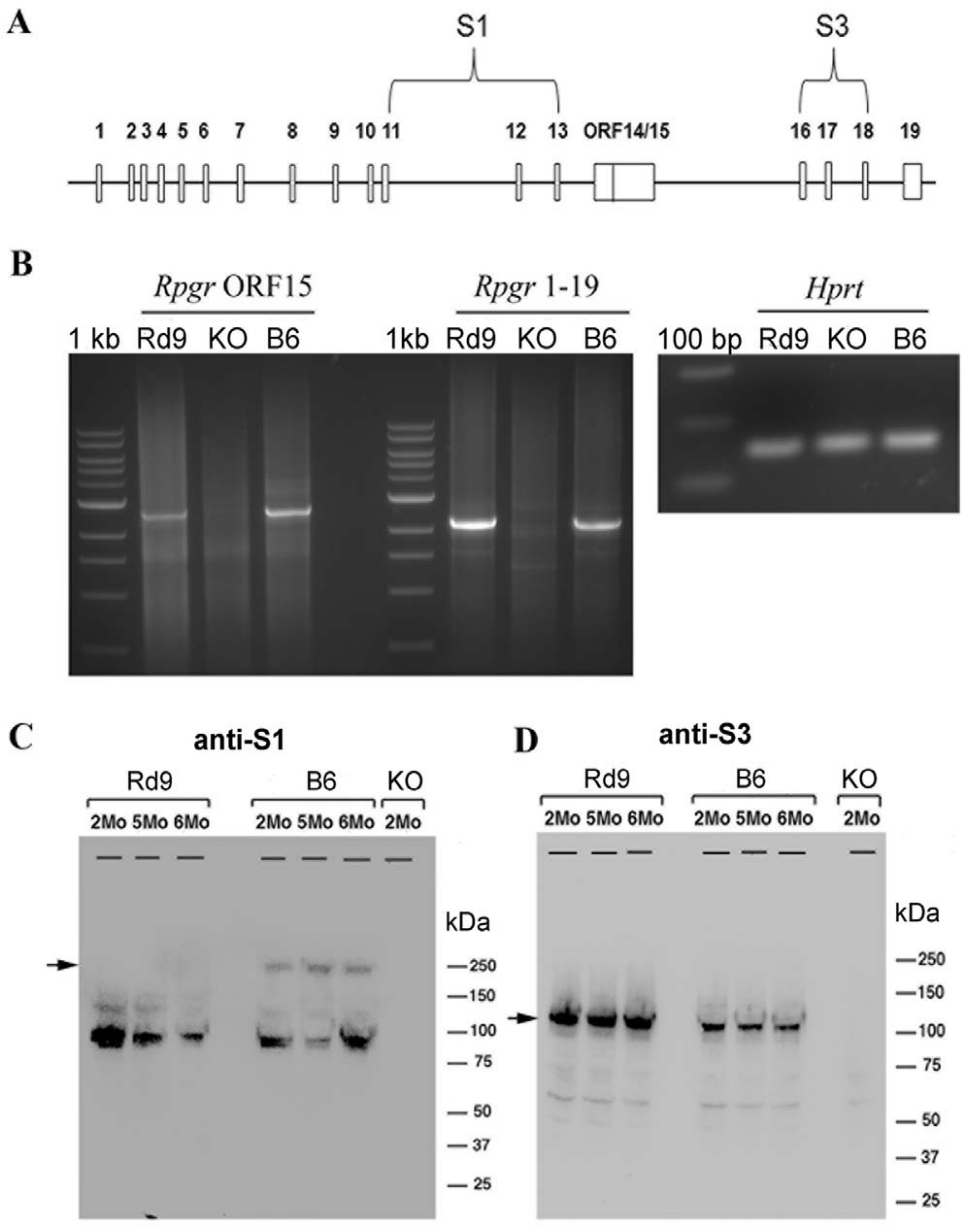
RT-PCR and immunoblot analysis of Rpgr expression in mutant and wild-type mice. **A**) Schematic of the mouse *Rpgr* gene showing the locations of the ORF15 exon, the oligonucleotide primers for RT-PCR, and protein sequences used to generate the RPGR antibodies S1 and S3. **B**) RT-PCR was performed on total retinal RNA from two-month-old male mice using a common exon 1 primer and reverse primers for ORF15 or exon 19. The PCR products were resolved on a 1% agarose gel. RT-PCR of *Hprt* was used as a control for RNA recovery and loading. **C**) Western blots of retinal extracts from 2-6 month-old male B6 (wild type), Rd9, and *Rpgr*-KO mice probed with RPGR antibodies. The S1 antibody recognizes a sequence common to both Rpgr ORF15 and 1–19 variants. The S3 antibody recognizes only the protein corresponding to the 1–19 variant.

RT-PCR analysis was performed using total retinal RNA from 3-month-old Rd9, B6, and *Rpgr*-KO mice to examine major alternatively spliced forms of *Rpgr* mRNA. Both Rd9 and B6 strains exhibited PCR products corresponding to *Rpgr* 1-ORF15 and exon 1–19 (Figure 2B). In contrast, these *Rpgr* transcripts appeared substantially reduced or absent from *Rpgr*-KO mice, as shown previously [26].

Immunoblot analysis was performed using retinal protein lysates from Rd9, B6, and *Rpgr*-KO mice at 2 and 6 months-of-age. The antibodies used recognized Rpgr epitopes shared by both variants (S1), or present in the constitutive variant only (S3) (Figure 2A) (16). In B6 mouse retina, the Rpgr-ORF15 isoform (≈250 kDa) was recognized by S1 antibody, whereas the constitutive Rpgr 1–19 isoform (≈98 kDa) was recognized by both S1 and S3 antibodies (Figure 2C). In retinal lysates from Rd9 mice, the Rpgr 1–19 isoform was present, but no Rpgr-ORF15 isoform was detected. Retinal extracts from Rpgr-KO mice showed no specific reactivity with either antibody, indicating the absence of both major Rpgr isoforms.

**Figure 3:**
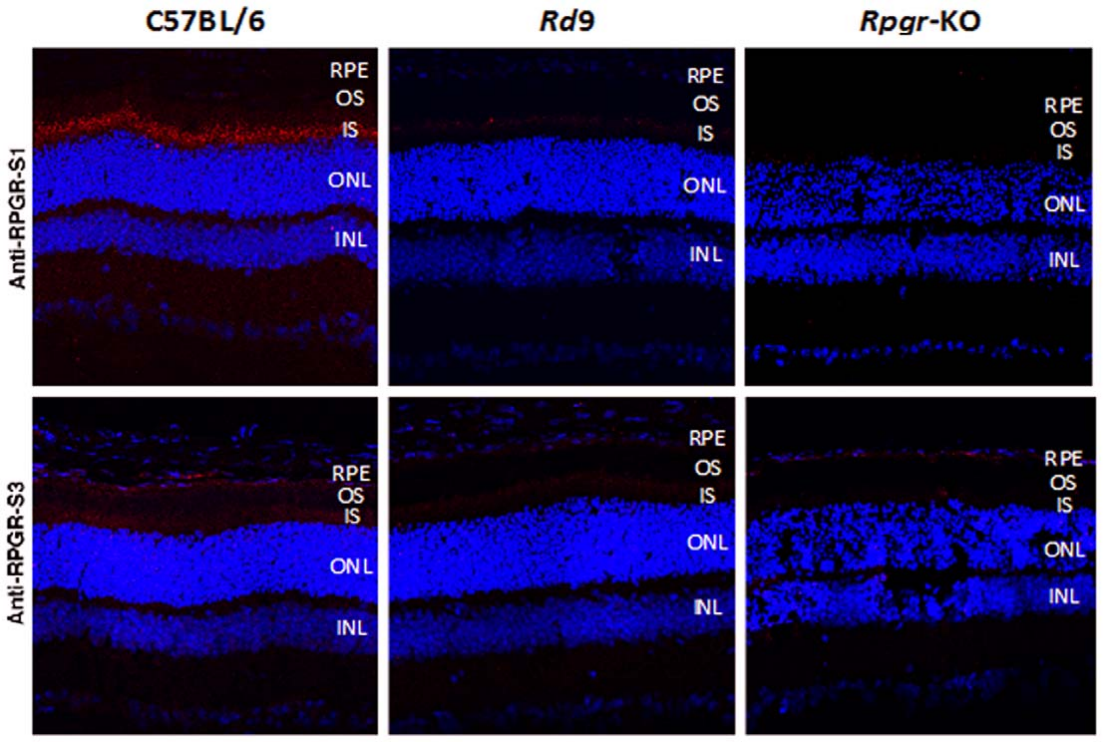
Immunohistochemical analysis of Rpgr in mutant and wild-type mice. Cryosections of eyes from 2-month-old B6, Rd9, and *Rpgr*-KO male mice were probed with S1 antibody that recognizes a sequence common to both Rpgr ORF15 and 1–19 variants, and S3 antibody that recognizes a sequence unique to the 1–19 variant. Red shows Rpgr-specific staining; blue shows DAPI staining of nuclei.

The expression of Rpgr variants was also investigated using immunohistochemistry on retina sections from 3-month-old Rd9/Y, B6, and *Rpgr*-KO mice. The S1 antibody that recognizes both constitutive and ORF15 isoforms showed strong immunostaining in the photoreceptor connecting cilium of B6 mice, but little reactivity was observed in Rd9 retinas (Figure 3). The S3 antibody that recognizes only the constitutive isoform showed low-level immunoreactivity in photoreceptor inner segments of B6 mice, and this staining was further reduced in Rd9 retinas. No S1 or S3 immunoreactivity was detected in *Rpgr*-KO retinas, as reported previously [26]. Our data are consistent with reports that the Rpgr-ORF15 isoform is present primarily in the photoreceptor connecting cilium, whereas the constitutive Rpgr 1–19 isoform is present in the cytosol [16], [32].

### Rd9 Retinal Histology

**Figure 4:**
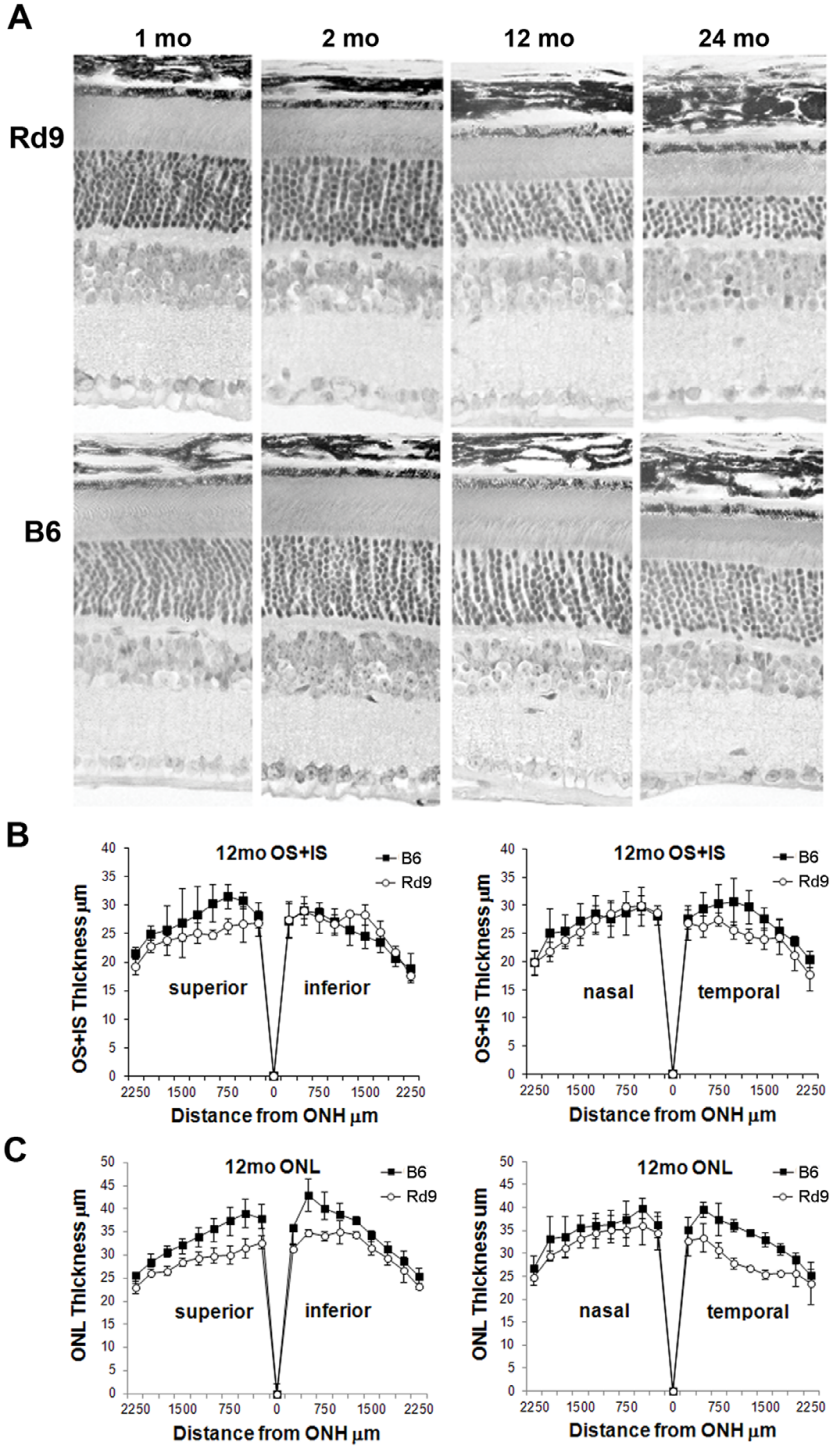
Analysis of retinal histology in Rd9 and wild-type mice. **A**) Photographs of plastic retina sections from Rd9/Y and B6 mice at the ages indicated. Morphometric analysis showing **B**) outer plus inner segment (OS+IS), and **C**) outer nuclear layer (ONL) thickness, in mice at 12 months-of-age. Thickness measurements were taken on sections parallel (superior/inferior) or orthogonal (nasal/temporal) to the vertical meridian of eyes from Rd9/Y (○) and B6 (▪) mice and plotted vs. distance from the optic nerve head, with standard deviations shown.

Retina sections from Rd9/Y mice showed largely normal structure and outer nuclear layer (ONL) thickness in animals 1 and 2 months-of-age (Figure 4A). For young animals, morphometric analysis demonstrated equivalent thickness of retinal cell layers in Rd9/Y and wild-type B6 mice, except for a slightly smaller combined length of outer plus inner segments (OS+IS) in the Rd9/Y superior and temporal regions (data not shown). This profile of OS+IS length persisted essentially unchanged up to 12 months-of-age (Figure 4B). Although the figures and data show a trend toward differences in the OS+IS thickness, overall analysis across all locations (by mixed regression) does not reach statistical significance (p = 0.07). During aging, Rd9/Y mice exhibited a gradual loss of ONL cell counts and overall decrease in photoreceptor layer thickness, while the inner retina appeared relatively well preserved. Morphometric analysis of ONL thickness in Rd9/Y mice at 12 months-of-age showed significant decreases in the superior, inferior, and temporal regions (p<0.05) (Figure 4C). By 24 months-of-age, the overall thickness of the photoreceptor layer in Rd9/Y mice was approximately half that at 1 month-of-age.

### Photoreceptor Protein Expression in Rd9

**Figure 5:**
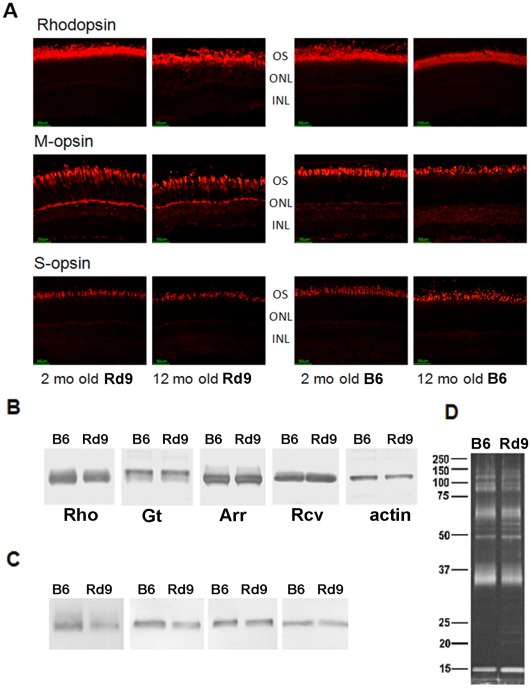
Rhodopsin and cone opsin expression in Rd9 and wild-type mice. **A**) Immunohistochemical analysis of M-opsin, S-opsin, and rhodopsin in the superior retina in Rd9/Y and B6 mice at 2 and 12 months-of-age (red fluorescence labeling). M-cone opsin reactivity appears throughout both outer and inner segments in Rd9 mice at both ages. Rhodopsin reactivity is disturbed in Rd9 mice at 12 months-of-age. **B**) Western analysis of rhodopsin, transducin-alpha subunit, arrestin, and recoverin in blots of total eye retinal protein homogenates from Rd9 and B6 mice at 1 month-of-age. The band corresponding to monomeric rhodopsin is shown, and β-actin served as a loading control. **C**) Immunoblot analysis of rhodopsin, transducin, arrestin, and recoverin in ROS preparations from Rd9 and B6 mice. **D**) SyproRuby staining of ROS proteins separated by SDS-PAGE verified equivalent loading of Rd9 and wild-type protein (β-actin, Hprt, and Gapdh were not informative markers for ROS preparations). Representative results are shown (n = 3). Rhodopsin (Rho), transducin-alpha subunit (GT-α), arrestin (Arr), and recoverin (Rcv).

Immunohistochemical analysis of rhodopsin expression in Rd9/Y mice at 2 months-of-age showed outer-segment labeling similar to that of wild-type B6 mice (Figure 5A). However, in Rd9/Y mice at 12 months-of-age, rhodopsin staining in the superior retina appeared diffuse and decreased in intensity, consistent with decreased ROS integrity and/or protein stability with aging. Extensive mislocalization of M-opsin within cones in the superior retina was seen in Rd9/Y mice as young as 2 months-of-age, with immunolabeling present throughout the cone inner segment, perinuclear and synaptic regions. Mislocalization of M-opsin in the inferior retina of Rd9/Y mice was evident in animals by 12 months-of-age (data not shown). In contrast, mislocalization of S-opsin (largely expressed in the inferior retina) was not seen in Rd9/Y mice up to 12 months-of-age, consistent with lesser disease involvement in the inferior hemisphere.

Immunoblot analysis of total retinal protein extracts from 1-month-old Rd9/Y and B6 mice showed equivalent levels of the phototransduction proteins, transducin, arrestin, and recoverin, but rhodopsin levels in Rd9/Y mice were reduced (Figure 5B). Rhodopsin levels also appeared lower in ROS preparations from Rd9/Y mice (Figure 5C), estimated by densitometry to be about half of those present in ROS from B6 mice in samples with equivalent protein loaded (Figure 5D).

### Age-dependent Changes in Retinal Function in Rd9

**Figure 6:**
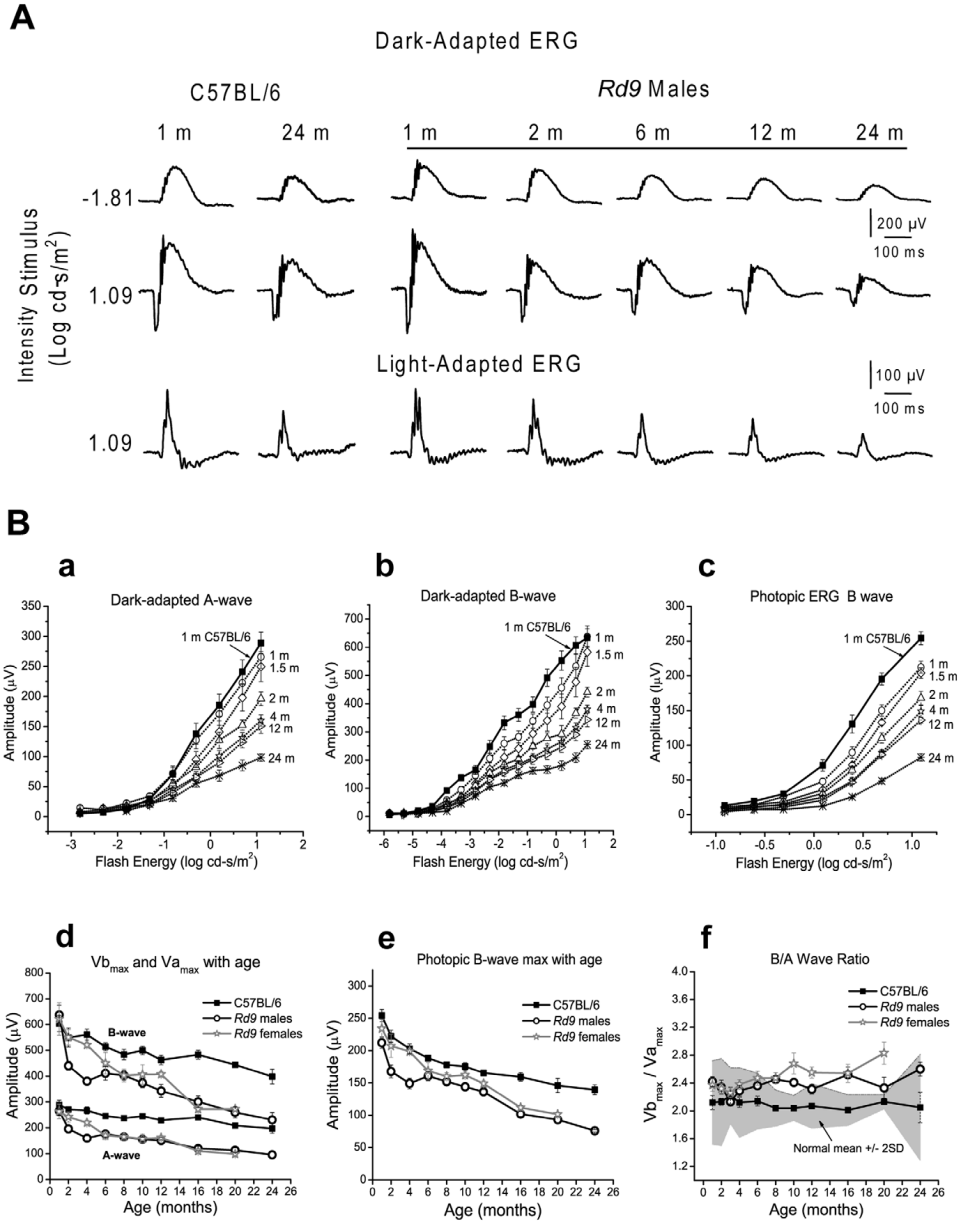
Electroretinogram (ERG) waveforms for Rd9 and wild-type mice. **A**) Representative dark-adapted and light-adapted ERGs waveforms. For mice at 1 to 24 months-of-age, dark-adapted ERG recordings show the rod-mediated responses and maximal responses (rod plus cone), and light-adapted ERG recordings show the maximal cone-mediated responses**. B**) ERG intensity-response function V-Log I curves in Rd9 males (open symbols) at 1 (n =  10), 1.5 (n = 6), 2 (n = 13), 4 (n = 11), 12 (n = 11), and 24 months (n = 5) compared to 1-month-old B6 mice (filled squares). **a**) dark-adapted a-wave. **b**) dark-adapted b-wave. **c**) photopic b-wave. Natural history of maximum ERG amplitude (Mean ± SE) changes in Rd9/Y males, Rd9/Rd9 females, and B6 mice. **d**) Dark-adapted Va_max_ and Vb_max_. **e**) Photopic L_max_. **f**) b/a wave ratio. Grey area represents the wild-type normal range.

ERG responses of Rd9 mice were similar in shape and timing to those of wild-type B6 mice (Figure 6A). Although ERG amplitudes decreased with age in Rd9/Y mice, characteristic waveforms were retained up to 24 months-of-age. Intensity-response plots of dark-adapted and light-adapted ERGs showed minimal increases in the a- and b-wave thresholds with age, and were comparable to age-dependent threshold variations observed in B6 mice up to 24-months-old (Figure 6B, graphs a, b, and c). To analyze the ERG responses of Rd9 and B6 mice, the rod photoreceptor-mediated negative going a-wave was measured from the baseline to its trough. The b-wave, which reflects bipolar cell activity, was measured from the a-wave trough to the peak of the b-wave. The dark-adapted ERG data were characterized by the rod saturated b-wave amplitude, V_max_, and response sensitivity, *k* derived from Naka-Rushton fits to the b-wave amplitude [40]. The mixed rod-cone response was characterized by the b-wave amplitude Vb_max_ and a-wave amplitude Va_max_ at maximum intensity of 1.09 log cd.s.m^–2^. The photopic ERG was characterized by the b-wave amplitude L_max_ at the maximum intensity. The ERG parameters plotted as a function of age showed that at 1 month-of-age, Rd9 responses were comparable to those of B6 mice. Subsequently, ERG amplitudes for Va_max_, Vb_max_, V_max_ (data not shown), and L_max_ showed a gradual, progressive decline (Figure 6B, graphs d and e). For statistical analysis, liner mixed regression was used to adjust for correlated measures within a mouse over time. On average, wild-type B6 mice had larger Vb_max_ than Rd9/Y mice and this difference increased with age (p = 0.0110). Va_max_ showed a similar difference that increased with age between B6 and Rd9/Y mice (p = 0.0250). The photopic b-wave amplitude L_max_ was larger in B6 mice (p<0.0001) and this effect did not change with age. Peak implicit time increased by 3 ms for Va_max_, and by 7 ms for Vb_max_ by 6 months-of-age in the Rd9/Y mice, but there was no significant difference in the parameter *k* or in implicit time at any age compared to B6 mice. Female Rd9/Rd9 mice showed a slightly slower rate of decline, but all amplitudes matched those of male Rd9/Y mice by 6 months-of-age. By 24 months-of-age, ERG amplitudes of Rd9/Y males were reduced by 65% from the values at 1 month-of-age. Effects of aging were also evident in the ERGs of wild-type mice, as the values of the response amplitudes obtained for B6 mice at 1 month-of-age were reduced by 35% in animals at 24 months-of-age [41].

### Rd9 Inner Retinal Function

The dark-adapted ERG a-wave primarily reflects the photoresponse of rod photoreceptors [42], [43], whereas the ERG b-wave reflects bipolar activity postsynaptic to rods [44]–[46]. Therefore, to assess inner retinal function and changes in visual signaling [47], the dark-adapted b-wave amplitude normalized to the a-wave amplitude (Vb_max_/Va_max_, or b/a-wave ratio) was determined at each age. The b/a-wave ratio of male Rd9/Y mice was within normal range up to about 6 months-of-age (Figure 6B, graph f ), and was at the higher end of the normal range up to 24 months-of-age, suggesting that both b-wave and a-wave amplitudes declined at a similar rate at younger age. Female Rd9/Rd9 mice had larger than normal b/a wave ratio at all ages. Vb_max_ was linearly correlated to Va_max_ (R = 0.95) (data not shown), indicating linearity of signal transfer from rods to cones as the degeneration progressed in Rd9/Y mice. Transient oscillatory potentials (OPs) that are driven by inner retinal activity [48] were present even in 24-month-old Rd9/Y mice, although the amplitude of the sum of OPs was reduced. To examine activity of the proximal retina, we measured the cone-driven photopic negative response (PhNR) [38] in Rd9/Y mice. The PhNR amplitude of Rd9/Y mice was consistently lower than in wild-type B6 mice at all ages and was down by 40% from the initial value at 1 month-of-age (data not shown).

### Correlation of Retinal Structure (disease) and Function

We used the relationship between the thickness of the photoreceptor layer and the ERG parameters Vb_max_ and Va_max_ to assess the stage of degeneration [49]. In Rd9/Y mice, the OS+IS and ONL thicknesses were concordant with the ERG amplitudes. The relationship between the ERG parameters Vb_max_ and Va_max_ and OS+IS thickness could be described by a linear function (Vb_max_ = –227.6+23.47*thickness, R =  0.96; and Va_max_ = –138.9+11.34*thickness, R = 0.95) (Figure S2). The slope of Vb_max_ was nearly double that of Va_max_ indicating that Vb_max_ is a more sensitive indicator of photoreceptor degeneration. We found similar linear relationships between ONL and ERG maximum amplitudes (Vb_max_ and Va_max_).

## Discussion

Our screening protocol identified Rd9 mice as a naturally-occurring model of human *RP3*, an X-linked retinal degeneration caused by mutations in *RPGR*. As in the majority of cases of *RP3*, and in a significant fraction of males with simplex RP [3], retinal degeneration in Rd9 mice is caused by a mutation in the alternatively-spliced exon ORF15. This exon encodes a protein domain that is unique to the RPGR isoform present in the connecting cilia of photoreceptors and features a 154 amino acid region (in B6 mice) composed almost entirely of EEEGEG repeats. This region is encoded by a similarly repetitive genomic DNA with a near absence of pyrimidines (7 pyrimidines in 462 nucleotides). The 32-bp duplication identified in the ORF15 region of Rd9 mice is predicted to alter the reading frame and lead to a truncated Rpgr-ORF15 protein in which the C-terminal 108 amino acids are out-of-frame. In contrast to the highly acidic nature of the normal reading frame, the amino-acid residues after the frame shift in Rd9 are predominantly basic. The ORF15 mutant protein appears to be unstable and is not detected in Rd9 retinal extracts or connecting cilia. Although wild-type levels of the constitutive isoform Rpgr 1-19 appear to be retained in Rd9 retinal lysates, the levels detected in Rd9 photoreceptor cells appear to be decreased, possibly due to reduced cellular functions associated with the more abundant ORF15 variant.

The retinal phenotype of Rd9 mice is relatively mild compared to that present in *RP3* individuals, yet it shares several important features in common with the human disease. These include the presence of retinal pathology and reduction of ERG function at early ages. Our analysis of the natural history and time course of disease progression in Rd9 mice show that rod and cone ERG amplitudes were reduced as early as 6 weeks-of-age in both male Rd9/Y and female Rd9/Rd9 mice, consistent with the expression of RPGR in both rods and cones. As we moved into new mouse quarters, we repeated the ERG analysis on eight males and four females for up to six months to further confirm the initial decreases in amplitude. Amplitude reductions in all ERG parameters were reproducible; however, the reduction from controls seen in the first four months was 25% compared to 35% in the initial study (Fig. 6B, graph d). Loss of amplitudes observed at young ages may be due to one or more mechanisms, including defects in photoreceptor OS and IS formation [50]. The decline in amplitudes occurring during aging correlated well with the reduction in ONL thickness, suggesting that photoreceptor cell loss is a contributing factor. Rd9/Y mice exhibited larger b/a-wave ratios compared to wild-type at later ages, suggesting that photoreceptor cell dysfunction predominates over inner retinal changes. Inner retinal abnormalities in *RPGR*-XLRP have been documented in humans [51] and in dogs [28] in the form of thickening of inner retinal structures secondary to ONL loss. Although we did not perform cell counts, the inner retina and ganglion cell layers of the Rd9/Y male mice showed no obvious loss by light microscopy.

The natural history of the Rd9 mouse is very similar to that previously reported for the *Rpgr*-KO mouse [26]. Unlike the *Rpgr*-KO mouse, the Rd9 retina retains the expression of the Rpgr 1-19 constitutive isoform. In both models, disease progression features rod pathology that develops progressively over a two year period, as well as cone opsin mislocalization that is evident from early ages onward. These similarities may be due to the fact that neither model expresses detectable Rpgr-ORF15 protein, albeit by different mechanisms. Unlike the *Rpgr*-KO mouse, the Rd9 retina retains the expression of the Rpgr 1–19 constitutive isoform. Thus, it appears that low level expression of the constitutive isoform in photoreceptors does not fully compensate for the absence of the ORF15 variant in Rd9 mice.

Mislocalization of rod and cone opsins is observed in post-mortem eyes from *RP3* carriers [52], [53], *XLPRA* dogs with two different mutations in ORF15 [28], and in two other genetically independent strains of *Rpgr*-mutant mice [26], [27]. For mice in which *Rpgr* exon 4 was deleted, genetic background was found to play a major role in determining the extent of rod and cone involvement, with rod disease predominating on the B6 background, and cone disease predominating on the albino BALB/cJ background [27]. Although cone-opsin mislocalization is an early hallmark of photoreceptor dysfunction associated with *RPGR* mutations, the underlying mechanism is not understood and appears likely to have multiple origins. In *Rpe65*-knockout mice, mislocalization of cone opsin was correlated to the absence of 11-cis retinal, and this defect could be corrected by administration of exogenous chromophore [54]. In *RPGR* mutant dogs, evidence of abnormal photoreceptor maturation resulting in abnormal outer segment structures was seen [50], a factor which could contribute to the decrease in rhodopsin levels such as those seen in young Rd9/Y mice. Rhodopsin labeling appears increasingly disrupted with aging, suggesting that *RPGR* mutations also impact rod outer segment stability. Studies of the recombinant ORF15 protein in *XLPRA2* indicate that rod-cone degeneration may result from aggregation of the mutant protein and its retention in the endoplasmic reticulum [28].

While there are no histopathologic studies of affected male patients with *RPGR* mutations, fundus abnormalities and histopathological reports in female carriers have shown a multifocal pattern of retinal dysfunction and disease, including opsin mislocalization and loss of photoreceptor nuclei [52], [53] similar to that seen in canine *XLPRA2* and *XLPRA1* carriers [55]. Autopsy eyes of three carrier individuals (age 75 to 86 years) revealed retinal pigment epithelium (RPE) abnormalities, including atrophy or proliferation, focal loss, and subretinal and intraretinal migration [56]. The autopsy eyes exhibited a patchy pattern of photoreceptor degeneration, and relative sparing of cones compared to rods, reduced or absent cone outer segments, and broad cone inner segments. Neurite sprouting from rods was also observed in a carrier with an *RPGR*-ORF15 Glu210 mutation. Human studies have also shown variable disease expression in female carriers, with some carriers of *RPGR* exon ORF15 mutations being severely affected while others manifest an asymmetric phenotype or are asymptomatic [57].

Clinical studies of males with XLRP due to mutations in *RPGR* show that patients have an earlier loss of visual acuity and visual fields compared to most other types of RP [58]. A recent study documented a wide range of clinical severity in 98 affected males [59]. Genetic factors such as allelic heterogeneity and genetic modifiers were shown to contribute to this diversity. Individuals with mutations in *RPGR* exons 1 to 14 have a more severe phenotype than those carrying ORF15 mutations [58]–[60]. In a large patient cohort followed over 8 years, ERG amplitudes declined 50% faster in individuals with mutations in exons 1–14 compared to those with ORF15 mutations; however, visual acuity and visual field loss were comparable in both groups [58]. Loss of acuity was attributed to photoreceptor cell loss detected by optical coherence tomography (OCT). In another study of two patients with *RPGR* mutations, *in vivo* microscopy revealed impaired rod, and well-preserved cone, function in early-stage disease, and absence of rod and cone function, and a deep foveal pit due to photoreceptor cell loss, in late-stage disease [61]. Our analysis of Rd9/Y male and Rd9/Rd9 female mice shows that disease caused by *Rpgr*-ORF15 mutations exhibits a uniform phenotype, beginning at very young ages and progressing gradually. In previous studies of the *RPGR* canine mutants, *XLPRA1* animals exhibited slow rod and cone photoreceptor degeneration, whereas *XLPRA2* animals exhibited abnormal photoreceptor development and rapid degeneration [28], [55]. Although the human phenotype is highly variable, disease onset generally occurs at a greater relative age and progresses rapidly compared to the animal models. Differences in the rates of animal and human development in early life, and inconsistencies between age-of-onset and age-of-diagnosis, however, make direct comparisons of disease severity relative to lifespan difficult.

XLRP caused by mutations in *RPGR*-ORF15 represents a significant portion of all inherited retinal degenerations, making it an important focus of basic and translational research. The disease in Rd9 mice appears to be milder than in individuals with *RP3*, but shares a number of important features in common that validate this naturally occurring mouse strain as an important model of the human disease. These include a frame-shift mutation in ORF15, mislocalization of opsin to the cone inner segments, reduction of ERG function at early ages, and progressive loss of ERG amplitudes with aging. We anticipate that the Rd9 model of XLRP will become a valuable addition to the tools available for analysis of RPGR function, associated disease pathogenesis, and the development of novel therapeutic strategies.

## Supporting Information

Figure S1 **Retina images.** A) Fundus photographs of wild-type and mice with the Rd9 genotypes indicated at 5 months-of-age. B) Following injection, fluorescein dye shows telangiectasia and some leakage from vessels in the Rd9 mice.
(TIF)

Figure S2 **Correlation of scotopic a-wave (Vamax) and b-wave (Vbmax) maximum amplitude with IS plus OS thickness in Rd9 mice.**
(TIF)

Table S1 **Primers used to PCR amplify and sequence mouse** ***Rpgr*** **.**
(DOC)
