# Supplementary figures and images for: Rd9 Is a Naturally Occurring Mouse Model of a Common Form of Retinitis Pigmentosa Caused by Mutations in RPGR-ORF15
Source: PLoS One. 2012 May 1;7(5):e35865. doi: 10.1371/journal.pone.0035865 (PMC3341386; doi:10.1371/journal.pone.0035865)

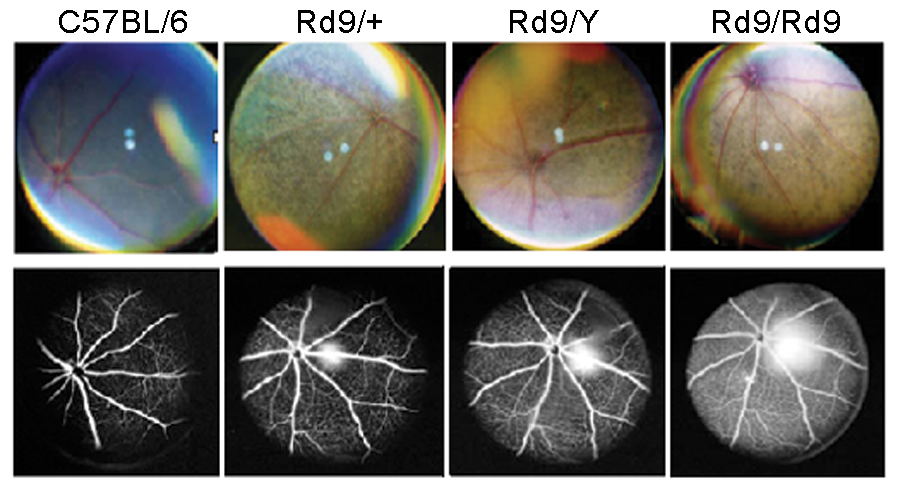

Supplement: Figure S1 — Retina images. A) Fundus photographs of wild-type and mice with the Rd9 genotypes indicated at 5 months-of-age. B) Following injection, fluorescein dye shows telangiectasia and some leakage from vessels in the Rd9 mice. (TIF) [file pone.0035865.s001.tif]

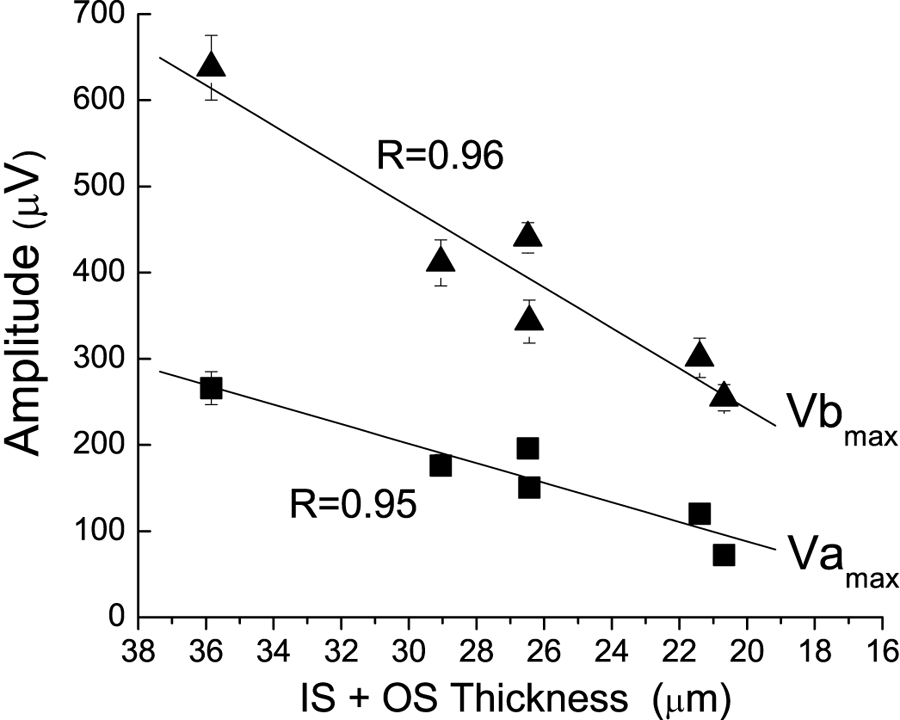

Supplement: Figure S2 — Correlation of scotopic a-wave (Vamax) and b-wave (Vbmax) maximum amplitude with IS plus OS thickness in Rd9 mice. (TIF) [file pone.0035865.s002.tif]
