# Supplementary material for: Rd9 Is a Naturally Occurring Mouse Model of a Common Form of Retinitis Pigmentosa Caused by Mutations in RPGR-ORF15
Source: PLoS One. 2012 May 1;7(5):e35865. doi: 10.1371/journal.pone.0035865 (PMC3341386; doi:10.1371/journal.pone.0035865)
Supplement: Table S1 — Primers used to PCR amplify and sequence mouse Rpgr . (DOC) [file pone.0035865.s003.doc]

**Table S1. Primers used to PCR amplify and sequence mouse *Rpgr***

| **Product** | **Forward** | **Reverse** |
| --- | --- | --- |
| **cDNA primers** |  |  |
| Exons 1-3 | GCGGAATCTGAGTCACTGGT | TTGGCTTGATGATAGCAGCTT |
| Exons 3-12 | TGGGGTCAGTTAGGATTAGGA | TTCTGTCTCTGTGTCTTTGTTCAT |
| Exons 1-19 | GCAGCACCTTAGGCTCAATC | CAGCAGAACCAACCAGACAT |
| Exon 3-14 | TGTTCGGCAGTAACAACTGG | GAATCTCGGCTGGTCGTATC |
| Exon 1 F | GGTCTATGCCAAGAGGGTCG |  |
| Exon 19 R |  | CCTGAAGATTTTGACCCATG |
| Exon 15 R |  | AGATCCGTCCTCTCTCTCCC |
| **Genomic primers** |  |  |
| ORF15 (RAP3/P7) | GAGAAAGTGATGGAAAGTACACCGTGCAC | GAGATGACTTCCCTGTTACTTCAATTCCAG |
| ORF 15 (F208) | ACGGGGATCAAATCTGTGAG |  |
| ORF15 (R1476) |  | TGTGCCATGTCTGCCATATT |
